# Nanobody peptide conjugate: a novel CD163 based broad neutralizing strategy against porcine reproductive and respiratory syndrome virus

**DOI:** 10.1186/s12951-024-02662-7

**Published:** 2024-07-02

**Authors:** Haotian Yang, Meiqi Sun, He Qiu, Huiling Xu, Zhuofan Deng, Han Gu, Nan Wang, Liuyang Du, Fushan Shi, Jiyong Zhou, Fang He

**Affiliations:** 1 MOA Key Laboratory of Animal Virology, Zhejiang University Center for Veterinary Sciences, Hangzhou, China; 2 Institute of Preventive Veterinary Medicine, College of Animal Sciences, Zhejiang University, Hangzhou, China; 3 ZJU-Xinchang Joint Innovation Centre (TianMu Laboratory), Gaochuang Hi-Tech Park, Xinchang, Zhejiang P.R. China; 4 Department of Veterinary Medicine, College of Animal Sciences, Zhejiang University, Hangzhou, 310058 Zhejiang China

**Keywords:** PRRSV, Antiviral peptides, Broad neutralization, Nanobody, Receptor binding domain

## Abstract

**Background:**

Porcine reproductive and respiratory syndrome virus (PRRSV) is a prevalent swine pathogen, which has caused adverse impact on the global swine industry for almost 30 years. However, due to the immune suppression caused by the virus and the genetic diversity in PRRSV, no virus-targeting broad neutralizing strategy has been successfully developed yet. Antiviral peptide and nanobody have attracted extensive attention with the ease in production and the efficacy in practice. In this study, four new fusion proteins named nanobody peptide conjugates (NPCs) were developed by combining PRRSV specific non-neutralizing nanobodies with CD163-derived peptides targeting the receptor binding domain (RBD) of PRRSV proteins.

**Results:**

Four NPCs were successfully constructed using two nanobodies against PRRSV N and nsp9 individually, recombining with two antiviral peptides 4H7 or 8H2 from porcine CD163 respectively. All four NPCs demonstrated specific capability of binding to PRRSV and broad inhibitory effect against various lineages of PRRSV in a dose-dependent manner. NPCs interfere with the binding of the RBD of PRRSV proteins to CD163 in the PRRSV pre-attachment stage by CD163 epitope peptides in the assistance of Nb components. NPCs also suppress viral replication during the stage of post-attachment, and the inhibitory effects depend on the antiviral functions of Nb parts in NPCs, including the interference in long viral RNA synthesis, NF-κB and IFN-β activation. Moreover, an interaction was predicted between aa K31 and T32 sites of neutralizing domain 4H7 of NPC-N/nsp9-4H7 and the motif 171NLRLTG176 of PRRSV GP2a. The motif 28SSS30 of neutralizing domain 8H2 of NPC-N/nsp9-8H2 could also form hydrogens to bind with the motif 152NAFLP156 of PRRSV GP3. The study provides valuable insights into the structural characteristics and potential functional implications of the RBD of PRRSV proteins. Finally, as indicated in a mouse model, NPC intranasally inoculated in vivo for 12–24 h sustains the significant neutralizing activity against PRRSV. These findings inspire the potential of NPC as a preventive measure to reduce the transmission risk in the host population against respiratory infectious agents like PRRSV.

**Conclusion:**

The aim of the current study was to develop a peptide based bioactive compound to neutralize various PRRSV strains. The new antiviral NPC (nanobody peptide conjugate) consists of a specific nanobody targeting the viral protein and a neutralizing CD163 epitope peptide for virus blocking and provides significant antiviral activity. The study will greatly promote the antiviral drug R&D against PRRSV and enlighten a new strategy against other viral diseases.

**Graphical abstract image:**

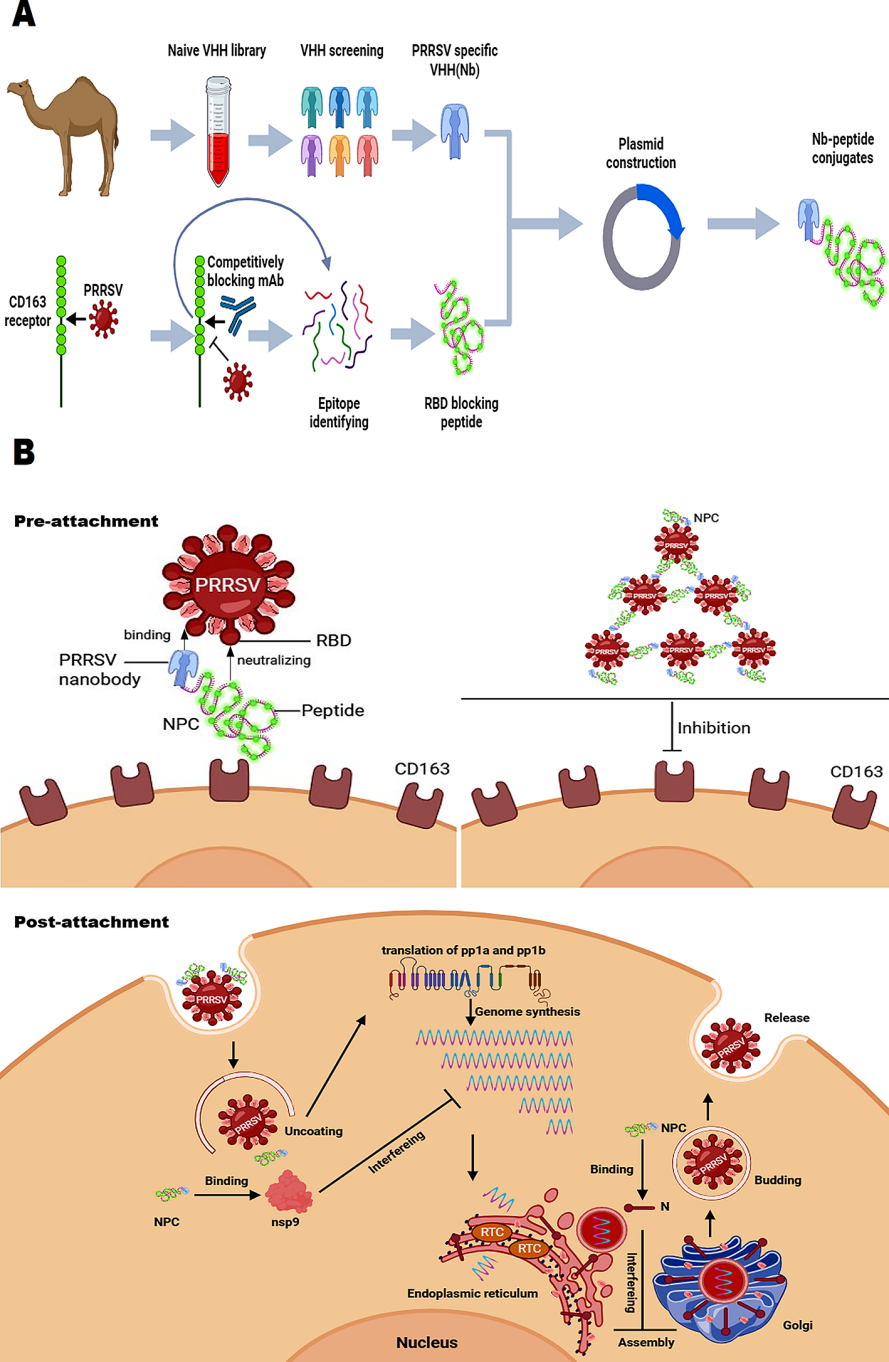

**Supplementary Information:**

The online version contains supplementary material available at 10.1186/s12951-024-02662-7.

## Introduction

Porcine reproductive and respiratory syndrome virus (PRRSV) is a highly contagious agent that causes late-term abortions and stillbirths in sows and respiratory disease in piglets [1]. PRRSV is an enveloped, single-stranded positive-sense RNA virus. The genome of PRRSV is approximately 15.4 kb in length, encoding seven structural proteins and at least 16 non-structural proteins (nsps). Structural proteins include nucleocapsid protein N, envelope protein E, membrane protein M, and glycoproteins (GPs) GP2a, GP2b (M), GP3, GP4, and GP5 [2]. PRRSV isolates have been divided into two genotypes: European-like isolates (Type 1, prototype Lelystad) and North American-like isolates (Type 2, prototype VR-2332) [3]. In China, Type 2 PRRSV is predominant and the diversity of the virus is increasing because of recombination events. The main strains prevailing in China include lineage 1 (NADC30-like), lineage 3 (QYYZ-like), lineage 5 (VR-2332-like), and lineage 8 (JXA1-like/CH-1a-like) [4].

PRRSV exhibits high genetic variability, resulting in significant antigenic differences among strains [5]. This complexity hampers the design and development of effective vaccines. PRRSV significantly compromises the immune defense by inhibiting the function of macrophages as well as the response of virus-specific T cells [6, 7], culminating in the impaired ability of immune system to recognize and eliminate the virus. Moreover, antibody-dependent infection enhancement (ADE) caused by the insufficient antibody-virus binding further increases the risk from those conventional vaccines at the low immunogenicity [8]. Though the commercially available modified live vaccine (MLV), such as Ingelvac PRRS^®^ MLV, has been used for almost three decades against PRRSV infection [9, 10]. More and more pig farms at the high safety standard, which are required to be free of PRRSV pathogen, are looking for alternatives beyond these live attenuated vaccines. Hence, antiviral agents such as peptides or antibodies other than vaccines, are emerging as the promising candidates for PRRSV prevention and control.

Nanobodies (Nbs) are variable domains of heavy chains of antibodies (VHHs) derived from Camelidae [11]. Nbs have several advantages over traditional antibodies, including the smaller size (15 kDa), the ease of genetic manipulation, the high stability and the solubility. Given these unique characteristics, Nbs serve as ideal candidate for drug development. Currently, specific Nbs have been identified against the PRRSV N and nsp9 [12, 13]. However, these Nbs inhibited virus intracellular replication but failed to block virus attachment extracellularly. Hence, a non-infection-permissive neutralizing nanobody against PRRSV is in the urgent demand.

Polypeptide drugs offer several advantages, including the stability, the ease of preparation, the safety and specificity. In recent years, researchers have conducted extensive researches to develop and optimize antiviral peptides. Antiviral peptides against various viruses, involving HIV [14, 15], influenza virus [16], hepatitis B virus [17] and coronavirus [18], have been successfully developed. These peptides played different roles during the infection through blocking virus binding or entry, interfering with intracellular transcription and inhibiting viral replication. Recently, receptor based antiviral strategies have been proved to be effective in blocking the binding between virus and the receptor, as reported in many virus studies, including SARS-COV-2 [19]. PRRSV relies on specific cellular receptors to infect host cells and complete the viral life cycle. The scavenger receptor CD163 plays a pivotal role in PRRSV infection by promoting viral uncoating and internalization in host cells [20]. Our previous study has developed two peptides from porcine CD163-SRCR5-9 as a highly effective strategy which can block the interaction between CD163 and viral glycoproteins to prevent PRRSV infection [21].

Given the high affinity to PRRSV of nanobodies and CD163 peptides, the combination of the two elements may offer a promising effective approach to address the above issues encountered in PRRSV control. In this study, we constructed potent and broad-spectrum inhibitors named nanobody peptide conjugates (NPCs), by linking existing non-neutralizing antibodies [12, 13] with neutralizing antiviral peptides. The binding and inhibitory activity against PRRSV were comprehensively characterized and evaluated. These results offer an alternative antiviral strategy against PRRSV and pave the way for the development of novel antiviral peptides in practical applications.

## Materials and methods

### Cell lines and viruses

PRRSV-permissive Marc-145 cells were cultured in Dulbecco’s modified Eagle’s medium (DMEM; Life Technologies Corp, Grand Island, NY, USA) supplemented with 10% fetal bovine serum (FBS; Gibco, Carlsbad, CA, USA) and penicillin-streptomycin (Life Technologies Corp, Grand Island, NY, USA). Porcine alveolar macrophages (PAMs) were obtained from 6-week-old PRRSV-negative pigs using a lung lavage technique and cultured in RPMI 1640 supplemented with 10% FBS and penicillin-streptomycin. All cells were cultured at 37 °C with 5% CO_2_.

The following PRRSV strains were used in this study: HuN4-F112 (GenBank ID: EF635006.1), JXA1 (GenBank ID: EF112445.1), HNXX-16 (GenBank ID: MH588709.1), JS18-3 (GenBank ID: MN606304.1), FH-112 (GenBank ID: EU480712.1), JK-100 (GenBank ID: AF332735.1). All PRRSV strains were propagated and titrated in Marc-145 cells or PAMs and stored at -80 °C.

### Protein expression and purification

Recombinant plasmids pET30a-Nb-N and pET30a-Nb-nsp9 were synthesized by Zhejiang Sunya Biological Technology Company. Gene fragments for Nb-N, Nb-NSP9, 4H7, and 8H2 were generated by PCR using pET30a-Nb-N, pET30a-Nb-nsp9, pGEX4T-4H7, pGEX4T-8H2 as the template and primers shown in Table 1. Linking Nbs to antiviral peptides by using linker(G4S)_3_. pET30a plasmid containing the sequences for a 6-histidine (6×His) tag upstream of the gene insertion site, was used as the expression vector. Plasmids named pet30a-Nb-N-4H7, pet30a-Nb-N-8H2, pet30a-Nb-nsp9-4H7, pet30a-Nb-nsp9-8H2, pet30a-Nb-N and pet30a-Nb-nsp9, were constructed by inserting the corresponding PCR fragments into pET30a plasmid. The inserted fragments were confirmed by sequencing. Plasmids were transformed into *Escherichia coli* BL21 (DE3), and protein expression was induced by 1 mM isopropyl β-D-thiogalactopyranoside (IPTG) at 16 °C for 24 h. Recombinant proteins in inclusion bodies were dissolved in 8 M urea for denaturation and then purified by Ni-NTA resin under denaturing conditions according to the manufacturer’s instructions. The denatured proteins were refolded by rapid dilution in base refolding buffer (880 mM L-arginine, 55 mM Tris, 21 mM NaCl, 0.88 mM KCl, pH 8.2) with 10 mM EDTA, 150 mM reduced glutathione, and 15 mM oxidized glutathione. For the subsequent cell experiments, the refolded proteins were dialyzed into 0.01 M phosphate-buffered saline (PBS). Collected samples were subjected to SDS-PAGE on 12% gel and protein was identified in western blot with 6×His mAb (Proteintech, China) diulted 1:1000 and stored at -80 °C until use.

The novel NPC consists of Nb-N CDR3 was synthesized and by Sango Biotech Company (China).

**Table 1:** Primers for expression plasmid construction

Plasmids	Primer sequences (5’ − 3’)	
pet30a-N-8H2	F	AGCACCACCACCACCACCACCAGGTGCAGCTGCAGGAAAGCG
	R	GCAGCCGGATCCTCGAGTTACGAGGATGATGAATTGCACGGG
pet30a-N-4H7	F	AGCACCACCACCACCACCACCAGGTGCAGCTGCAGGAAAGCG
	R	GCAGCCGGATCCTCGAGTTAGTCCCAGTGAGAGTTGCAGAGGGACC
pet30a-nsp9-8H2	F	AGCACCACCACCACCACCACCAGGTTCAGCTGCAGGAAAGCGG
	R	GCAGCCGGATCCTCGAGTTACGAGGATGATGAATTGCACGGG
pet30a-nsp9-4H7	F	AGCACCACCACCACCACCACCAGGTTCAGCTGCAGGAAAGCGG
	R	GCAGCCGGATCCTCGAGTTAGTCCCAGTGAGAGTTGCAGAGGGACC
pet30a-NPC(NC)	F	AGCACCACCACCACCACCACCAGGTGCAGCTGGTTGAAAGTG
	R	GCAGCCGGATCCTCGAGTTAATGGTGATGATGATGATGATGGTGATGATGATG
pet30a-N	F	AGCACCACCACCACCACCACCAGGTGCAGCTGCAGGAAAGCG
	R	GTTAGCAGCCGGATCCTCGAGTTAGCTGCTCACGGTCACCTGGGT
pet30a-nsp9	F	AGCACCACCACCACCACCACCAGGTTCAGCTGCAGGAAAGCGG
	R	GCAGCCGGATCCTCGAGTTAGCTGCTCACTGTAACCTGGGTACCCTGA
pet30a-vector	F	GTGGTGGTGGTGGTGGTGCTTGTC
	R	TAACTCGAGGATCCGGCTGCTAACAAAGC
pet30a-N-8H2	F	CTCATCTCTGAAGAGGATCTGCAGGTGCAGCTGCAGGAAAGCG
	R	CTTATCATGTCTGGATCCCCCTACGAGGATGATGAATTGCACGGGGATA
pet30a-eGFP	F	TAAAAGCTTGCGGCCGCACTCGAGG
	R	CTTGTACAGCTCGTCCATGCCGAGA CTCGGCATGGACGAGCTGTACAAGCAGGTTCAGCTGCAGGAAAGCGGT
pet30a-eGFP-NSP9-4H7	F	GAGTGCGGCCGCAAGCTTTTAGTCCCAGTGAGAGTTGCAGAGGGACC
	R	CTCATCTCTGAAGAGGATCTGCAGGTGCAGCTGCAGGAAAGCG
pCMV-N-4H7	F	CTCATCTCTGAAGAGGATCTGCAGGTGCAGCTGCAGGAAAGCG
	R	TCTTATCATGTCTGGATCCCCCTAGTCCCAGTGAGAGTTGCAGAGGG
pCMV-nsp9-8H2	F	CTCATCTCTGAAGAGGATCTGCAGGTTCAGCTGCAGGAAAGCGGTG
	R	CTTATCATGTCTGGATCCCCCTACGAGGATGATGAATTGCACGGGGATA
pCMV-nsp9-4H7	F	CTCATCTCTGAAGAGGATCTGCAGGTTCAGCTGCAGGAAAGCGGTG
	R	TCTTATCATGTCTGGATCCCCCTAGTCCCAGTGAGAGTTGCAGAGGG
pCMV-NPC(NC)	F	CTCATCTCTGAAGAGGATCTGCAGGTGCAGCTGGTTGAAAGTGGTGGCGG
	R	TCTTATCATGTCTGGATCCCCCTAATGGTGATGATGATGATGATGGTGAT
pCMV-vector	F	TAGGGGGATCCAGACATGATAAGATACAT
	R	CAGATCCTCTTCAGAGATGAGTTTCTG

### Cell viability and cytotoxicity assay

The cell viability and cytotoxicity associated with NPCs were evaluated using a Cell Counting Kit-8 (CCK-8; Beyotime Institute of Biotechnology, Shanghai, China). Briefly, Marc-145 cells or PAMs were seeded into 96-well cell culture plates and cultured in 10% FBS + DMEM at 37 °C in 5% CO_2_ overnight. The old culture medium was then replaced with new 2% FBS + DMEM/RPMI 1640 containing different concentrations of NPCs, then continued to culture for different periods. 10 µL of CCK-8 reagent was added to each well containing 100 µL of DMEM and incubated at 37 °C for 2 h. The absorbance of each well at 450 nm was detected using an epoch microplate.

### Virus neutralization

To verify the function of NPCs on PRRSV infection, 200 TCID_50_ of PRRSV (JXA1, HuN4-F112, JS18-3, HNXX-16, FH-112, JK-100) was mixed with peptides at various concentrations at 37 °C for 1 h. After incubation, 100 µl of the peptides-virus mixture was added to Marc-145 cells or PAMs in each well of 96-well plate at 37 °C for 1 h. At 24 hpi, cells were collected and tested for PRRSV N protein expression level by IFA. 200 TCID_50_ of PRRSV (JXA1, HuN4-F112, JS18-3, HNXX-16, FH-112, JK-100) was mixed with NPCs (100 µg/ ml) at 37 °C in each well of 48-well plate at 37℃ for 1 h. Cells were collected and total RNA was extracted for qRT-PCR 24 hpi. Cells were treated by the same process, collecting the supernatants for viral titers detection at 72 hpi.

### Attachment and internalization assay

For virus attachment assay, HuN4-F112 strain was mixed with an equal volume of NPCs with various concentrations at 4 °C for 2 h, and the mixture was added to 96-well plates at 4 °C for 2 h to allow virus attachment. For virus internalization assay, HuN4-F112 strain was added to chilled cells on ice for 2 h. After rinsed with ice-cold PBS for four times, cells were incubated with NPCs on ice for 1 h and then shifted to 37 °C for 2 h. After internalization, cells were washed with Hanks solution for four times to remove unbound virus. Cells were further resuspended in 200 µL trypsin-EDTA (Gibco, Grand Island, NY, USA) and proteinase K (Sungene, Shanghai, China) for 5 min, and washed for four times with Hanks solution to remove unbound virus. Finally, cells were collected and tested for PRRSV by qRT-PCR.

### Western blot

NPCs were collected and subjected to RIPA lysis buffer (Beyotime, Shanghai, China) for protein extraction. The extracted protein samples were separated by 12% SDS-PAGE and subsequently transferred to PVDF membranes. The membranes were then blocked with 5% (w/v) skimmed milk in PBST for 2 h at room temperature. Following blocking, the membranes were incubated with anti-6×HIS monoclonal antibody at 37 °C for 1 h. After thorough rinsing with PBST five times, the blots were incubated with HRP-coupled goat anti-mouse IgG (diluted at 1:5000, Sangon Biotech, China) at 37 °C for 1 h. Subsequently, the membranes were rinsed five times, and the immunoreactive signals were detected using the Super Signal West Pico/Femto chemiluminescent substrate.

### Indirect immunofluorescence assay (IFA)

Cells were fixed with 4% paraformaldehyde (Sigma-Aldrich) at -20 °C for 30 min and then washed three times with PBS. Subsequently, the cells were incubated with mouse 6×His antibody or VH13 for 1 h at room temperature, followed by three washes with PBS. Cells were then incubated with HRP-conjugated goat anti-mouse IgG (H + L) for 1 h at room temperature. After three additional washes with PBS, the cells were observed. with RPMI-1640 medium containing 10% FBS after 1 h-incubation. Virus replication was identified in IFA and virus titer was calculated according to Reed-Muench method.

### Quantitative real-time PCR

Total RNA from cells was extracted using the TRIzol reagent (Vazyme Biotech, NanJing, China) following the provided instructions from the manufacturer. Reverse transcription and qPCR were performed using a PrimeScript RT reagent Kit (Vazyme Biotech, NanJing, China) as previously described. The transcripts of β-actin or GAPDH were amplified and used as internal controls to normalize the total RNA input. The relative expression levels of the target genes were then quantified using the 2^−ΔΔCt^ method. Primers used in qPCR are shown in Table 2. 

**Table 2:** Primers for quantitative PCR (qPCR) analysis

Target genes	Primer sequences (5’ − 3’)	
PRRSV N	F	CCTCTAGCGACTGAAGATGACGTCAGGCATCACT
	R	ACTCCACAGTGTAACTTATCCTCCCTGAATCT
Monkey β-actin	F	ATCTGGCACCACACCTTCTACAATGAGCTGCG
	R	CGTCATACTCCTGCTTGCTGATCCACATCTG
Pig GAPDH	F	CCTTCCGTGTCCCTACTGCCAAC
	R	GACGCCTGCTTCACCACCTTCT
NF-κB	F	AGCAGATGGCCCATATCTTCA
	R	ATGGGATGGGCCTTCACAAA
IFN-β	F	GCAATTGAATGGAAGGCTTGA
	R	CAGCGTCCTCCTTCTGGAACT
5’UTR-F	F	CACCTTGCTTCCGGAGTTG
gRNA-R	R	GAGAGACCGTGCACTGAGACATC
sgRNA2-R	R	CAGCCAACCGGCGATTGTGAA
sgRNA3-R	R	GCAAAGCGGGCATACCGTGT
sgRNA4-R	R	ACGAAGTCTGATGCTGCGGTG
sgRNA5-R	R	CTGGCGTTGACGAGCACAGCA
sgRNA6-R	R	CATCACTGGCGTGTAGGTAATGGA
sgRNA7-R	R	GGCTTCTCCGGGTTTTTCTTCCTA

### Animal experiments

Animal experimental procedures were supervised and approved by Laboratory Animal Care and Use Committee at Zhejiang University. Guideline for Ethical Approval of Laboratory Animal Care (GB/T 35,892–2018), Guiding Opinions on Laboratory Animal Management Regulations (2006–398) (2017) were followed. The operating procedures for animal experiments were approved by the Laboratory Animal Management Committee of Zhejiang University.

Eight-week-old female BALB/c mice were randomly divided into 4 groups, NPC (12 h), PBS (12 h), NPC (24 h), PBS (24 h), respectively (*n* = 10). Mice were administrated intranasally of 200 µL NPC(N-4H7) (1 mg/mL) or PBS. Mice were sacrificed by cervical dislocation, then trachea and lung were collected at 12 and 24 h post administration. Tissues collected were minced and incubated with 200 µL DMEM containing PRRSV HuN4-F112 (10^3^ TCID_50_/mL) at 37 °C for 1 h. After filtering and centrifuging by centrifuge style filter (Costar^®^ Spin-X^®^), the supernatant (50 µL) was added to single-layer Marc-145 cells of a single well in 96-well-plate and replaced with DMEM medium containing 2% FBS after 1 h incubation. Cells lysates were collected for qRT-PCR and viral titer determination.

### Statistical analysis

Data were obtained from at least three independent experiments for the quantitative analysis and were expressed as means ± standard errors of the means. All statistical analysis were performed with t test or one-way analysis of variance (ANOVA). Asterisks *, **, *** or **** in figures indicate statistical significance at the *P* < 0.05, *P* < 0.01, *P* < 0.001, or *P* < 0.0001 level, respectively.

### Prediction of protein-protein complex structure

The protein sequences of PRRSV GP2a (GenBank ID: ABR26250.1), GP3 (GenBank ID: ABO68986.1), GP4 (GenBank ID: ABO68987.1), N (GenBank ID: QIN91219.1), nsp9 (GenBank ID: AID23859.1) were obtained from Genebank. Then, the 3D model of the protein was constructed using Alphafold 2. Finally, the predicted protein-protein complex structure models were visualized using Pymol 2.5 software.

## Results

### Expression and purification of NPCs

**Fig. 1:**
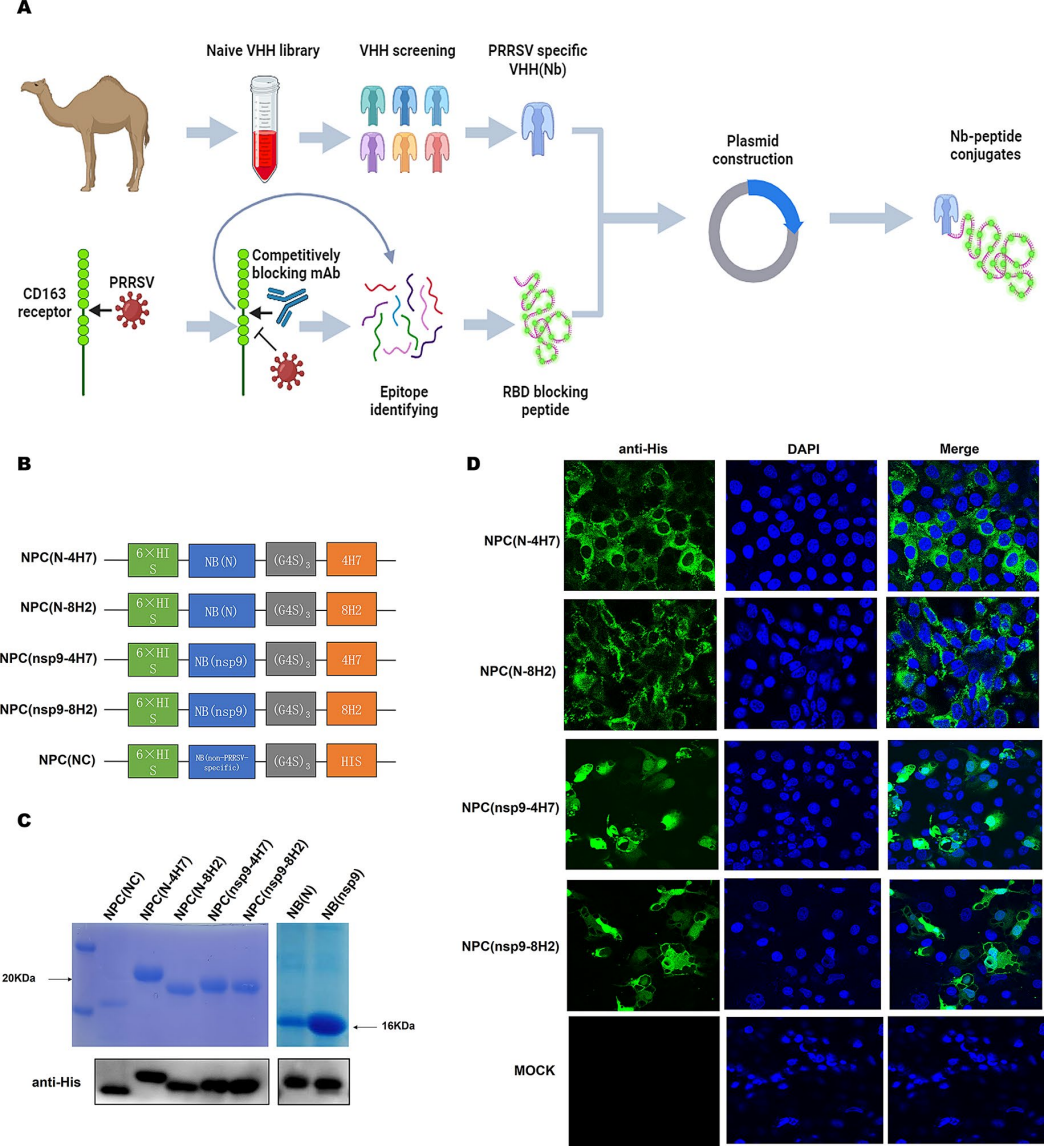
Expression and purification of NPCs. Schematic presentation of nanobody screen against PRRSV N protein and nsp9 from naive VHH library and the construction of nanobody peptide conjugates (NPCs) **(A)**. Schematic diagrams of different plasmids for 4 NPCs **(B)**. Construction of NPCs expression system using pET30a vector. Expression of NPCs and Nbs in *Escherichia coli* BL21 (DE3) were induced by IPTG. After purification using a Ni-column, the purified proteins were subjected to SDS-PAGE analysis and Western blot detection **(C)**. Detection of the binding activity of PRRSV with NPCs. Marc-145 cells were collected at 24 h post infection. Cells were fixed and permeabilized, then incubated with NPCs. Binding activity was determined with different NPCs (the primary antibody) and employed mouse anti-His antibody (the secondary antibody) as the secondary antibody, then probed with Alexa-Fluor-488 conjugated Goat anti-Mouse IgG (green) in confocal microscopy **(D)**

Four PRRSV-specific nanobody peptide conjugates (NPCs), including NPC(N-4H7), NPC(N-8H2), NPC(nsp9-4H7) and NPC(nsp9-8H2), were synthesized by linking two nanobodies targeting the PRRSV N/nsp9 to two peptides 4H7/8H2 derived from porcine CD163 targeting the RBD of PRRSV proteins. At the same time, a control NPC(NC), consisting of a non-PRRSV-specific nanobody and a multiple histidines peptide, was designed as a negative control (Fig. 1B). The above NPCs were cloned into expression vector pET-30a, and further transferred into *Escherichia coli* BL21 (DE3) strain. As shown in Fig. 1C, in SDS-PAGE and Western blot, the expressions of NPCs were identified at the molecular weight of about 16–20 kDa. NPCs were purified to high purity using Ni-resin purification. To further investigate the ability of NPCs binding to PRRSV, infected Marc-145 cells were fixed and incubated with NPCs which were used as primary detection antibody in IFA. As shown in Fig. 1D, all the four PRRSV-specific NPCs developed significant fluorescence signals in PRRSV infected cells. The results demonstrate that the four virus-specific NPCs can specifically bind to PRRSV.

### Purified NPCs inhibit PRRSV infection in a dose dependent manner

**Fig. 2:**
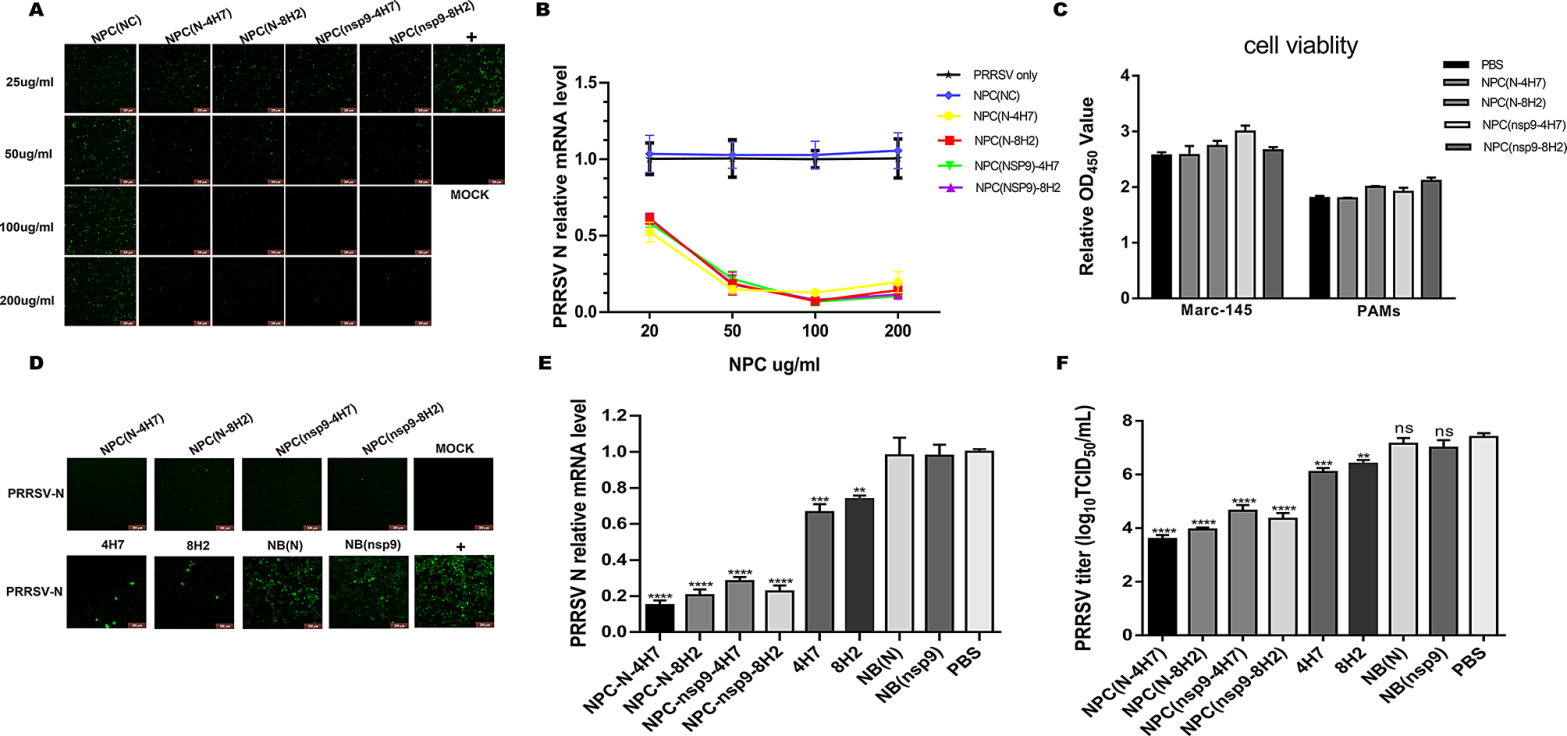
NPCs block PRRSV infection in Marc-145. The NPCs in various concentrations were incubated with 200 TCID_50_ PRRSV (HuN4-F112) at 37 °C for 1 h. Then the mixture was added to single-layer Marc-145 cells and replaced with DMEM medium containing 2% FBS after 1 h incubation. Virus replication was identified in IFA **(A)** and qRT-PCR **(B).** Four NPCs were testified using Cell Counting Kit-8 (CCK-8) assay to observe whether they have cell toxicity after adding to Marc-145 cells or PAMs at the concentration of 100 µg/mL after 24 h incubation **(C).** NPCs at a concentration of 50 µg/mL significantly inhibited the replication of PRRSV. Marc-145 cells were preincubated with 50 µg/mL NPCs, Nbs, CD163 peptides for 1 h, followed by the inoculation with PRRSV HuN4-F112. Cells were collected at 24 hpi and virus replication was identified in IFA **(D)**, qRT-PCR **(E).** Cell supernatants were used to detect viral titers at 72 hpi **(F)**. Experiments were performed in triplicate and data were shown as mean ± SD. Statistical significance is indicated as * (*P* < 0.05); ** (*P* < 0.01); *** (*P* < 0.001); **** (*P* < 0.0001)

To determine the specificity of NPCs inhibition against PRRSV, the irrelevant NPC control was tested together in a dose-gradient evaluation. Marc-145 cells were mock-infected or infected with 200 TCID_50_ of PRRSV, and treated with or without NPCs at various concentrations of 25, 50, 100 and 200 µg/mL. At 24 hpi, cells and supernatants were collected to determine the inhibitory effects of NPCs on PRRSV. A significant reduction in PRRSV N protein expression and transcription levels was observed under the treatment with any of virus-specific NPCs in a dose dependent manner, as compared with the NPC(NC) treatment cells (Fig. 2A&2B). Cell Counting Kit-8 (CCK-8) assay was performed to test the toxicity of NPCs in Marc-145 cells and PAMs. As shown in Fig. 2C, the viability of different types of cells was close to that of the PBS treated cells at concentrations of 100 µg/mL, which was used in further studies. Meanwhile, to compare the function of NPCs with the original peptides or Nbs, the inhibitory effect of NPCs at the concentration of 50 µg/mL on PRRSV infection was further confirmed through IFA, qRT-PCR and virus titer determination. Interestingly, at the same concentration of 50 µg/mL, the virus-specific NPCs successfully suppressed virus infection, as indicated with N protein expression (Fig. 2D), transcription levels (Fig. 2E), as well as viral titers (Fig. 2F), while limited inhibition was detected with peptides or Nbs treated alone. These results indicate that NPCs exert improved anti-viral activity as compared to either Nbs or peptides alone.

### NPCs provide broad inhibition against various PRRSV lineages

**Fig. 3:**
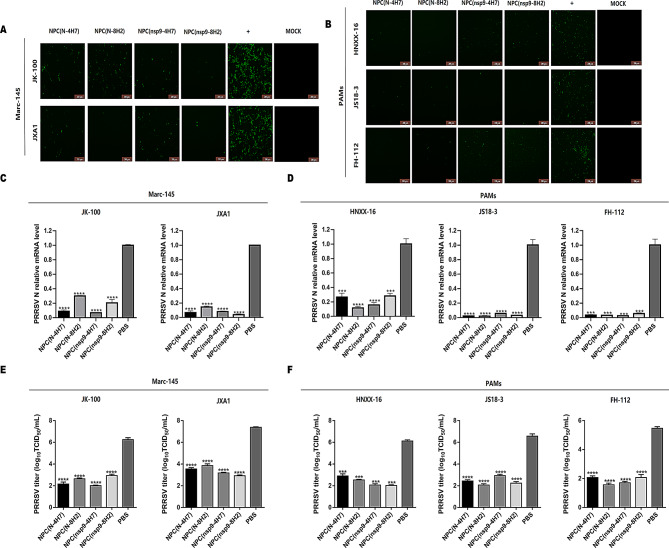
NPCs provide broad inhibition against various PRRSV strains. PAMs and Marc-145 cells were preincubated with 100 µg/mL NPCs for 1 h, followed by the inoculation with PRRSV strains individually, including JK-100, JAX1, HNXX-16, JS18-3, FH-112. Cells were collected at 24 hpi, and virus replication was identified in IFA **(A&B)** and qRT-PCR **(C& D).** Cell supernatants were used to detect viral titers at 72 hpi **(E& F)**. Relative changes of mRNA were compared to the PRRSV infection group without NPCs treatment. β-actin and GAPDH were detected as the internal reference in Marc-145 cells and PAMs respectively. Experiments were performed in triplicate and data were shown as mean ± SD. Statistical significance is indicated as * (*P* < 0.05); ** (*P* < 0.01); *** (*P* < 0.001); **** (*P* < 0.0001)

The potentialities of NPCs in universal protection were evaluated by different PRRSV lineages. Marc-145 cells treated with or without NPCs at 100 µg/mL were mock-infected or infected with 200 TCID_50_ of PRRSV strains (JK-100 strain of lineage 5, JXA1 strain of lineage 8). The inhibitory effect of NPCs was further confirmed in IFA, qRT-PCR and virus TCID_50_. As shown in Fig. 3, fluorescence signals of PRRSV N were not observed in groups treated with NPCs at 24 hpi (Fig. 3A). qRT-PCR results showed that the inhibition rate on PRRSV genome transcription of different lineages was > 60% in the group treated with NPCs, as compared with the PBS group (Fig. 3C). Furthermore, viral titers were decreased by 3–4 logs at 72 hpi with NPC treatment but not with PBS (Fig. 3E). These results indicate NPCs can significantly inhibit PRRSV infection in Marc-145 cells.

The efficiency of PRRSV suppression of NPCs was further evaluated in pulmonary alveolar macrophages (PAMs), the primary targets of PRRSV infection in vivo. Three different representative PRRSV strains including HNXX-16 strain of lineage 1, JS18-3 and FH-112 strains of lineage 8 were selected for the study. As shown in Fig. 3B, significant loss in fluorescence intensity of PRRSV N was detected in groups with NPCs. Meanwhile, PRRSV N mRNA levels of various PRRSV strains were also detected in qRT-PCR. The results showed that the inhibition rate by NPCs was > 70% as compared to control groups (Fig. 3D). And the results of viral titers indicated NPCs exerted more than 1000-fold inhibitory activity on different PRRSV strains as compared to PBS (Fig. 3F). In conclusion, these results confirm that NPCs display broad antiviral activity against different PRRSV strains from various lineages in both PAMs and Marc-145 cells.

### NPCs disturb PRRSV attachment, but the inhibition on post-attachment depends on the nanobody activity

**Fig. 4:**
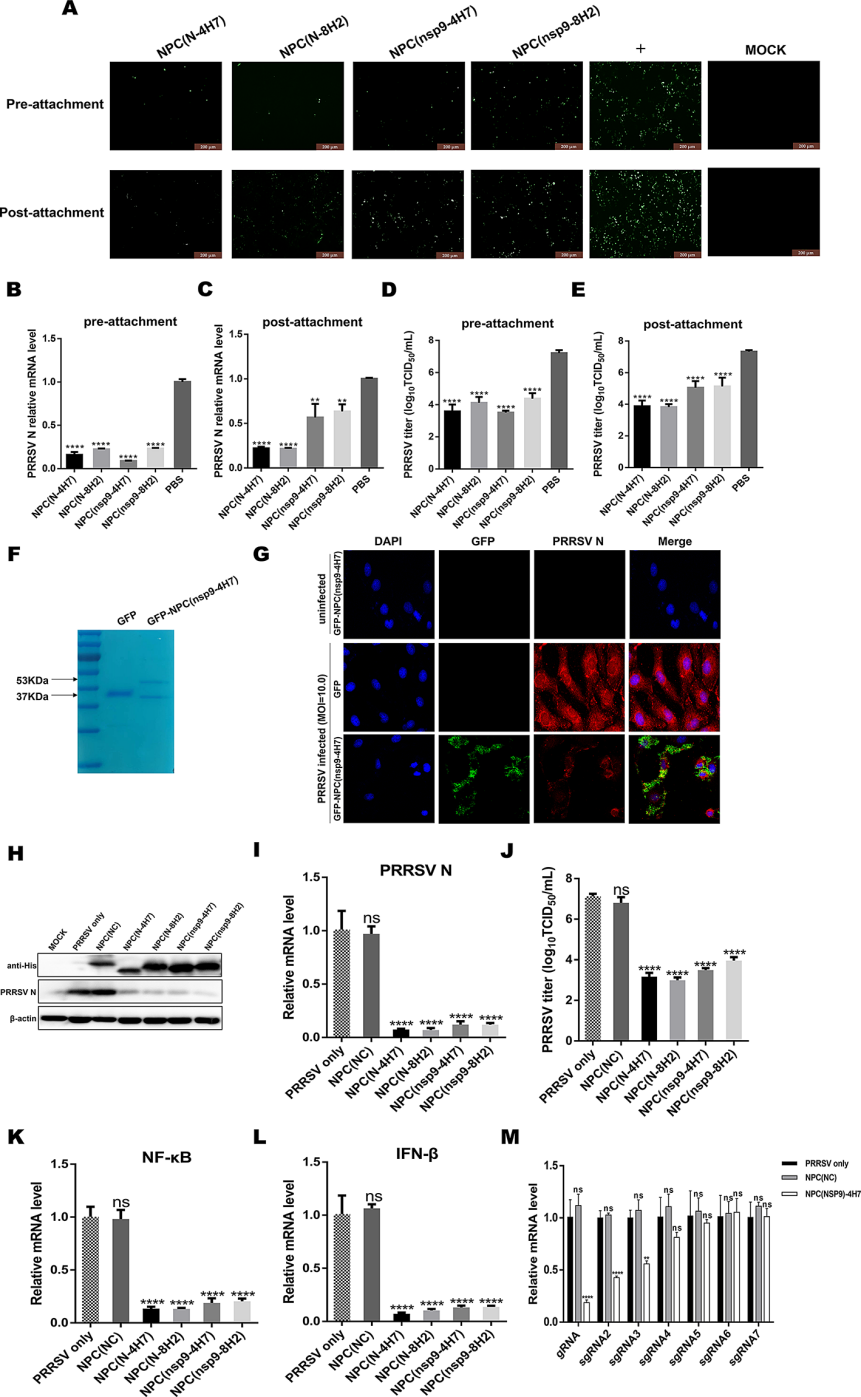
NPCs disturb PRRSV infection in both pre/post-attachment stage. For pre-attachment assays, cells were pre-incubated with NPCs (100 µg/mL) at 37 °C for 1 h, and inoculated with 200 TCID_50_ HuN4-F112 for 1 h. For post-attachment assays, cells were treated with HuN4-F112, and then post-incubated with NPCs. After 24 hpi, virus replication was identified in IFA **(A)**, total RNA was extracted and used as template for qRT-PCR **(B&C)** using primers specific for N. Cell supernatants were used to detect viral titers at 72 hpi **(D&E)**. Expression of GFP and GFP-NPC(nsp9-4H7) in *Escherichia coli* BL21(DE3) were induced by IPTG. After purification using a Ni-column, the purified proteins were subjected to SDS-PAGE **(F)**. Detection of the entry of GFP-NPC(nsp9-4H7) following with PRRSV (MOI = 10.0) into Marc-145 cells. Cells were collected at 12 hpi. Then cells were incubated with the anti-PRRSV N antibody (the primary antibody), and probed with Alexa-Fluor-594 conjugated Goat anti-Mouse IgG (the second antibody, red) in confocal microscopy **(G)**. To elucidate the antiviral mechanism of NPC in the post-attachment stage, the levels of PRRSV N were detected in western blotting **(H)** and qRT-PCR **(I)** at 24 hpi in NPCs transfected-Marc-145 cells. Cell supernatants were used to detect viral titers at 72 hpi **(J)**. Meanwhile, innate immunity response induced by infection was evaluated by the levels of NF-κB and IFN-β in qRT-PCR **(K&L)**. Quantification of the viral RNAs after overexpression of NPC(nsp9-4H7) was detected in qRT-PCR using specific primers **(M)**. Experiments were performed in triplicate and data were shown as mean ± SD. Statistical significance is indicated as * (*P* < 0.05); ** (*P* < 0.01); *** (*P* < 0.001); **** (*P* < 0.0001)

To elucidate the mechanism of NPCs antiviral activity, efforts were made to find out which step in virus infection was disturbed by NPCs. NPCs were inoculated to Marc-145 cells either prior to or post virus attachment. As depicted in Fig. 4A&4B, it was observed that NPCs at a concentration of 100 µg/mL prior to virus attachment led to a significant reduction in N protein fluorescence intensity and transcription level. This observation was consistent to the receptor-blocking function of the CD163 peptides [21], confirming antiviral function of NPCs in the stage of pre-attachment mainly relied on the peptide part. Furthermore, in the post-attachment stage, the successful suppression in PRRSV N expression and transcription was detected in all NPCs groups (Fig. 4A&4 C), though two NPCs-nsp9 presented mild inhibition, suggesting that NPCs targeting nonstructural protein served intracellular inhibition. The results of viral titers were consistent with the findings above (Fig. 4D&4E). Moreover, to visualize that NPCs were internalized in cells with virus, recombinant GFP control and GFP-NPC (nsp9-4H7) were cloned into expression vector pET-30a, and further purified to high purity using Ni-resin purification (Fig. 4F). In the post-attachment stage, PRRSV HuN4-F112 strain (MOI = 10.0) infected or mock infected Marc-145 cells were incubated with GFP or GFP-NPC (nsp9-4H7). As shown in Fig. 4G, at 12 hpi, as compared with GFP treated or uninfected group, significant green fluorescence signals (GFP-NPC) were detected in PRRSV infected cells incubated with GFP-NPC(nsp9-4H7), indicating GFP-NPC successfully enter cells via binding to PRRSV. Moreover, the intensity of red fluorescence signals (PRRSV N) was reduced or even abolished in GFP-NPC (nsp9-4H7) treated cells as compared with GFP control group, indicating an inhibition on virus replication upon NPC internalization. These results demonstrated that NPCs can specifically bind to PRRSV and exert antiviral activity in both the stages of pre- and post-attachment.

To further elucidate the inhibitory machinery of NPCs in the post-attachment stage, NPCs were overexpressed by transfection in Marc-145 cells followed by PRRSV HuN4-F112 infection (MOI = 1.0). PRRSV N was significantly decreased in the virus-specific NPCs transfected group in both protein and mRNA levels, as well as viral titers (Fig. 4H to 4J). Meanwhile, NPCs successfully suppressed infection-induced innate immunity response, as evaluated by the transcriptional levels of NF-κB and IFN-β, as compared with the control NPC (Fig. 4K&4 L). These results demonstrate that these NPCs have the activity to inhibit viral intracellular replication. The nanobody targeting PRRSV N protein has been demonstrated it can block the self-interaction of N protein following viral assembly [22]. Here, we explored the inhibitory mechanism of NPCs targeting nsp9 in the post-attachment stage. Quantification of the viral RNAs showed that overexpression of NPC(nsp9-4H7) markedly decreased the relative levels of long viral RNAs, as compared with NPC(NC). While no significant change was found in the synthesis of short sgRNA4, sgRNA5, sgRNA6 and sgRNA7 (Fig. 4M). These results indicate that NPCs have the capability to inhibit PRRSV infection both prior to and post virus attachment. Effectively blocking the CD163 receptor prior to virus attachment leads to a significant reduction in PRRSV invasion. Moreover, NPCs also have antiviral effects, which depends on the nanobody activity during the post-attachment stage.

### NPC with nanobody-CDR3 domain sustains effective antiviral activity

**Fig. 5:**
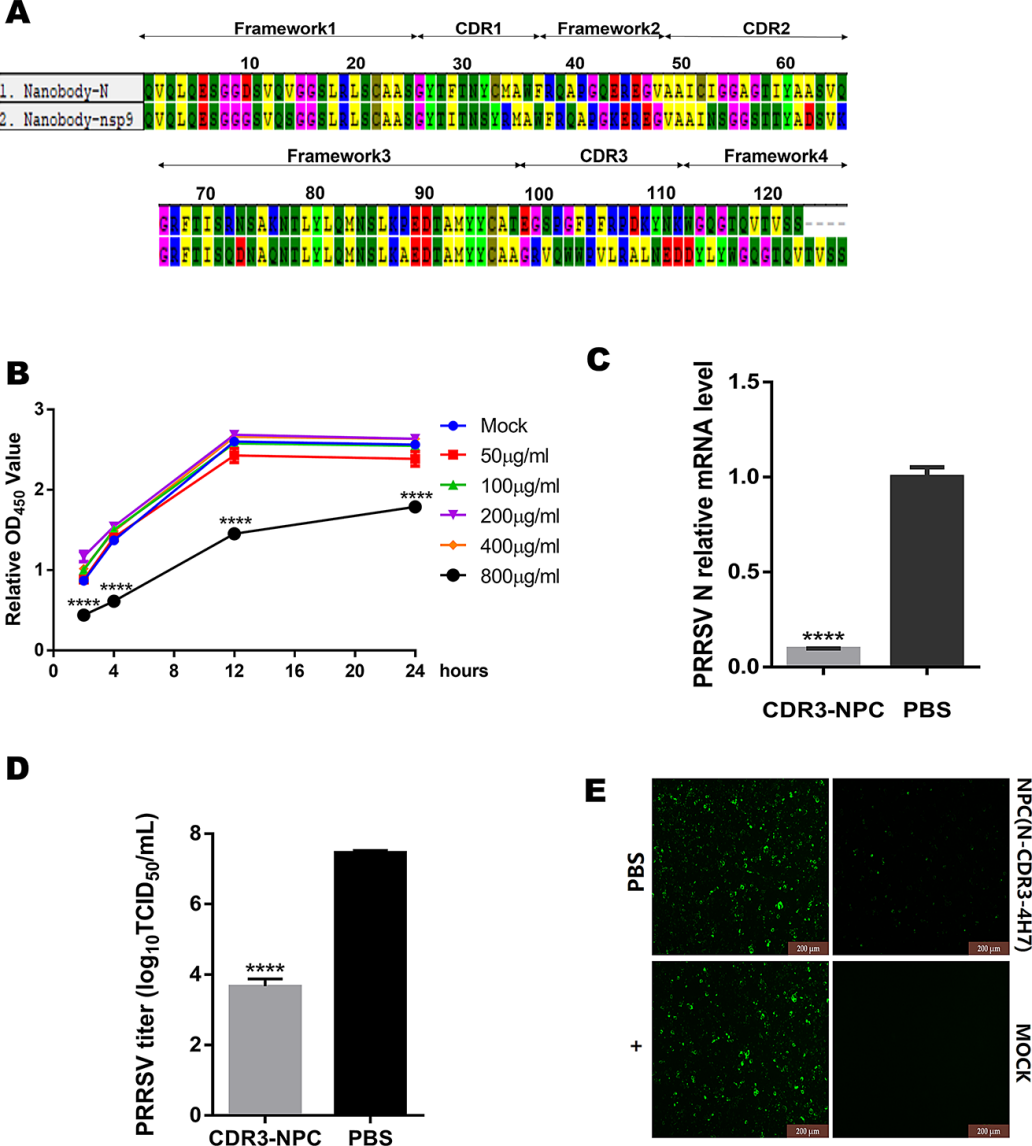
A novel shorter NPC which was synthesized from the nanobody CDR3 domain and CD163 epitope peptide 4H7 shows effective antiviral activity. An illustration of the structures of two nanobodies **(A).** The cell toxicity of novel smaller NPC was tested by Cell Counting Kit-8 (CCK-8) assay after adding to Marc-145 cells in different concentrations via measuring the absorbance at 450 nm using a microplate reader at different periods. **(B)** Next, the new NPC (100 µg/ml) was incubated with 200 TCID_50_ PRRSV (HuN4-F112) at 37 °C for 1 h. The mixture was added to single-layer Marc-145 cells and replaced with DMEM medium containing 2% FBS after 2 h-incubation. Virus replication was identified in qRT-PCR **(C)**, virus titer determination assay **(D)** and IFA **(E)**. Data were shown as mean ± SD. Statistical significance is indicated as * (*P* < 0.05); ** (*P* < 0.01); *** (*P* < 0.001); **** (*P* < 0.0001)

CDR3 region of nanobody has been proven to be the most crucial area for recognizing antigens (Fig. 5A). CDR3 of the Nb targeting PRRSV N, consisting 15 amino acids only, was directly linked to 4H7 peptide, creating a simplified NPC. No obvious cytotoxicity from the CDR3 NPC was detected from the concentration of 50 µg/mL to 400 µg/mL in Marc-145 cells (Fig. 5B). The antiviral activity of the CDR3 NPC was further studied at the same concentration (100 µg/mL) used in the full-length NPC. Interestingly, the CDR3 NPC also shown strong inhibition activity on PRRSV. The treatment with CDR3 NPC led to the inhibition rate of > 90% for PRRSV HuN4-F112 compared with PBS as indicated by PRRSV N mRNA level (Fig. 5C). Meanwhile, virus titer was decreased by more than 1000 folds (Fig. 5D) and suppressed fluorescence intensity from PRRSV N (Fig. 5E) was observed in IFA upon the CDR3 NPC treatment, confirming the antiviral activity of CDR3 NPC. Taken together, the results demonstrate that the simple CDR3 NPC retains the sufficient activity to suppress PRRSV as the full-length NPC, paving way for the cost-effective veterinary application.

### NPCs link PRRSV blockers against multiple targets for significant and broad neutralization

**Fig. 6:**
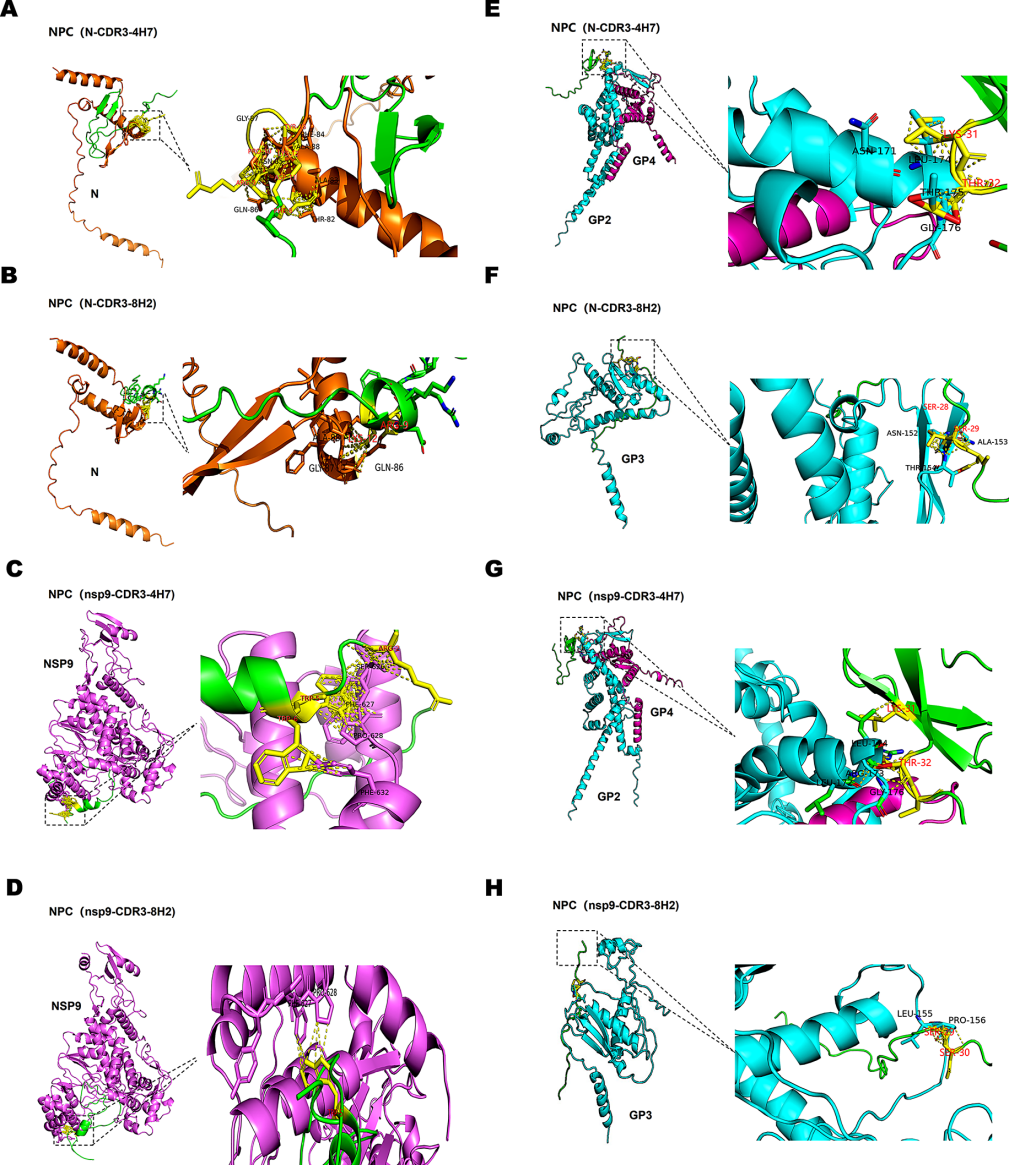
NPCs link PRRSV blockers against multiple targets for significant and broad neutralization. Three-dimensional models of the complex of NPCs and different PRRSV proteins which are responsible for virus binding to CD163 receptor or specific nanobody. The interacting residues are highlighted in black dotted boxes and colored in yellow. NPC(N-CDR3-4H7) binds to PRRSV N/GP2a + GP4 complex **(A&E)**. NPC(N-CDR3-8H2) binds to PRRSV N/GP3 **(B&F)**. NPC(nsp9-CDR3-4H7) binds to PRRSV nsp9/GP2a + GP4 complex **(C&G)**. NPC(nsp9-CDR3-8H2) binds to PRRSV nsp9/GP3 **(D&H)**

To understand the mechanism of NPCs specifically binding to PRRSV, the complex structures of NPCs and PRRSV N or nsp9 were predicted using Alpha Fold 2 (https://alphafold.com) and visualized by Pymol 2.5 software (https://pymol.org). As shown in Fig. 6A&6B, the results suggested a potential physical interaction forming by hydrogens, between the motif 8FRPDKY12 of nanobody-N CDR3 domain of NPC-N-CDR3-4H7/8H2 and the motif 82TAFNQGA88 of PRRSV N. In Fig. 6C, an interaction forming by hydrogens was indicated between aa R2, the W5 and W6 sites of nanobody-nsp9 CDR3 domain of NPC-nsp9-CDR3-4H7/8H2 and the motif 626SFP628 and the F632 site of PRRSV nsp9. Simultaneously, the model predicted in Fig. 6D also showed that the W5 site of nanobody-nsp9 CDR3 domain forms hydrogens to bind to aa F627 and P628 sites of PRRSV nsp9, indicating that W5 could be a vital site of nanobody-nsp9 CDR3 to recognize PRRSV nsp9.

Structural proteins GP2a, GP3, GP4, GP5 and M are known to play a crucial role in PRRSV infection. Among them, GP2a and GP4 function as a heterodimer. To further clarify the mechanism of NPCs neutralizing the virions, the complex structures of NPCs and PRRSV proteins which are responsible for virus binding to host receptor were also analyzed. There is no interaction predicted between NPCs and PRRSV GP4, GP5 or M. As shown in Fig. 6E&6G, the predicted interaction was indicated forming by hydrogens between the aa K31 and T32 sites of neutralizing domain 4H7 of NPC-N/nsp9-CDR3-4H7 and the motif 171NLRLTG176 of PRRSV GP2a. As for the neutralizing domain 8H2 of NPC-N/nsp9-CDR3-8H2, the motif 28SSS30 was found to contribute to bind with the motif 152NAFLP156 of PRRSV GP3 via forming hydrogens (Fig. 6F&6 H). These results confirm that, by combining PRRSV specific nanobodies with CD163-targeting antiviral peptides, NPCs achieve precise virus recognition and efficient neutralization. The combination further improves the virus inhibitory activity of two components as compared to the individual one in solo.

### NPC intranasally administrated in vivo sustains significant antiviral activity

**Fig. 7:**
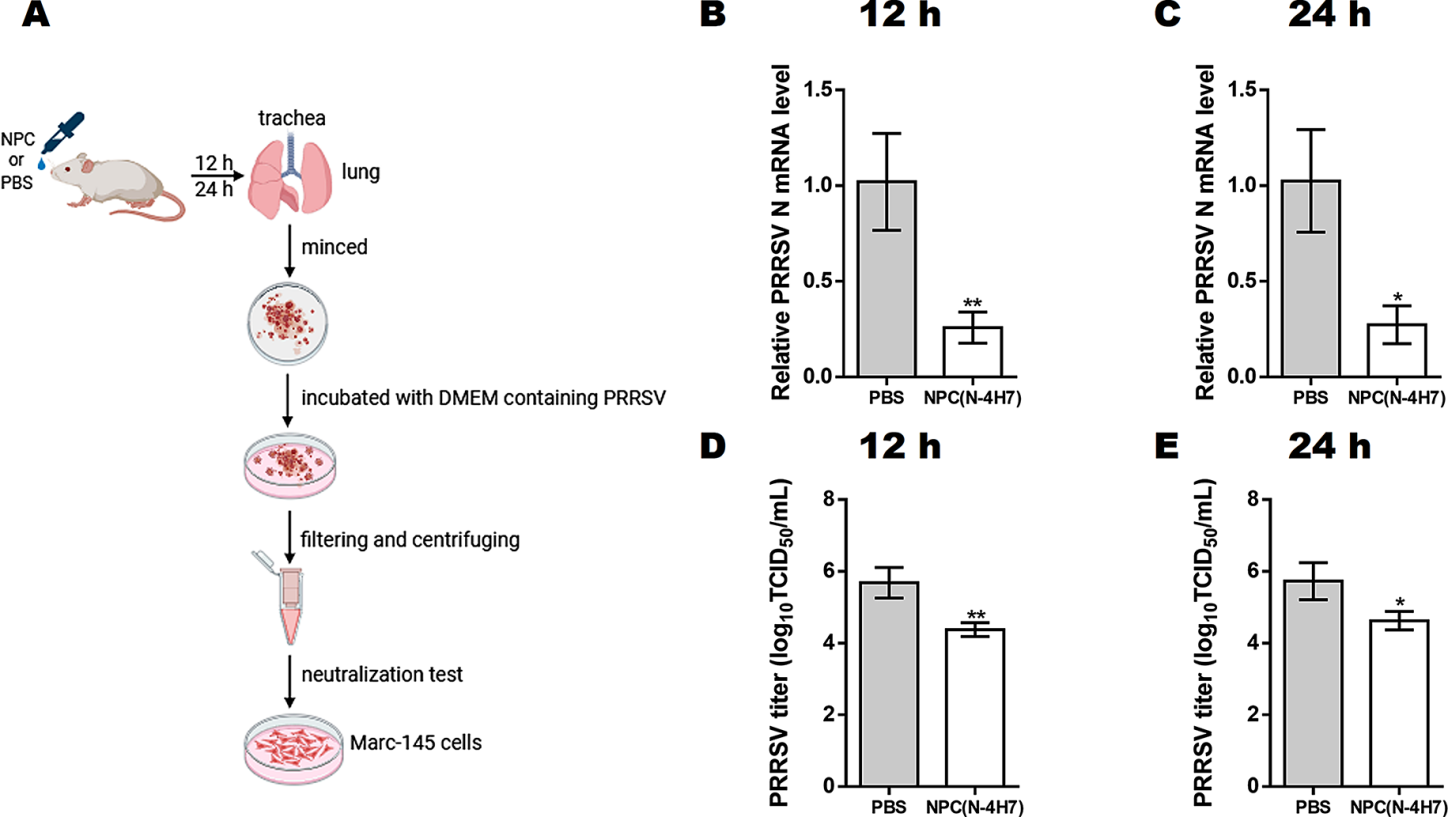
NPC intranasally administrated in mice sustains significant antiviral activity in Marc-145 cells. **(A)** An illustration of the animal experiment. Eight-week-old female mice were randomly divided into 4 groups, NPC (12 h), PBS (12 h), NPC (24 h), PBS (24 h), respectively (*n* = 10). Mice were administrated by intranasal delivery of 200 µL NPC(N-4H7) or PBS. Trachea and lung tissues were was collected at 12 and 24 h post administration. Tissues collected were minced and incubated with 200 µL DMEM containing PRRSV HuN4-F112 (10^3^ TCID_50_/mL) at 37 °C for 1 h. The filtrated supernatant was added to single-layer Marc-145 cells and replaced with DMEM medium containing 2% FBS after 1 h incubation. Virus replication was identified in qRT-PCR **(B and C)**, virus titer determination assay **(D and E)**. Data were shown as mean ± SD. Statistical significance is indicated as * (*P* < 0.05); ** (*P* < 0.01); *** (*P* < 0.001); **** (*P* < 0.0001)

To evaluate the neutralizing activity of NPC upon administration in vivo, a mouse model for intranasal inoculation was exploited and combined with virus neutralization in Marc-145 cells (Fig. 7A). Trachea and lung tissues of mice administrated with either NPC(N-4H7) or PBS were collected at 12 and 24 h post administration. The neutralizing activity of NPC in the collected tissues was evaluated against PRRSV infection in Marc-145 cells. As shown in Fig. 7B and C, compared with PBS group, intranasally delivered NPC shown the inhibition rate of > 60% against PRRSV HuN4-F112 at both 12 and 24 h post administration, as indicated by PRRSV N mRNA level. Meanwhile, virus titer was decreased by more than 100 folds (Fig. 7D and E). Overall, the results demonstrate that NPC intranasally administrated in vivo sustains the significant antiviral activity against PRRSV and suggest the potential of NPC to prevent respiratory infection from airborne viruses like PRRSV.

## Discussion

At present, PRRSV continues to be prevalent and causes extensive economic loss to the pig industry worldwide. Due to the features of the virus to escape from host immunity, the existing vaccines to control PRRS have limitations to provide complete protection without the risk of virus shedding and recombination [23]. Unfortunately, the recombinant events between field and MLV vaccine strains [24–26] occur frequently, together with the continuous genetic mutations. Hence, antiviral agents besides vaccines are emerging as alternative strategies against PRRSV, including miRNAs [27], antibodies and chemical compounds, such as tetrahydroaltersolanol C, N-acetylpenicillamine and dipotassium glycyrhetate [28]. Among these, bioengineered neutralizing antibody (NA) is still considered as one of the best antiviral biologic agents. Nevertheless, the progress in developing effective and broad NA against PRRSV is currently sluggish due to the high variance of PRRSV strains and the low neutralizing immunogenicity of PRRSV structural antigens [29]. Successful PRRSV NA production was rarely reported, impeding the aid in the control of PRRS. Nanobodies targeting nsp9 [30] and nsp4 [31] were identified and tested for antiviral activity against PRRSV, However, they exerted intracellular inhibition only and failed to stop virus infection in the pre-attachment stage. The blocking of infection is the key feature of typical NA, further improvements are required for these anti-PRRSV candidates to be converted into broadly and effectively neutralizing agents.

**Fig. 8:**
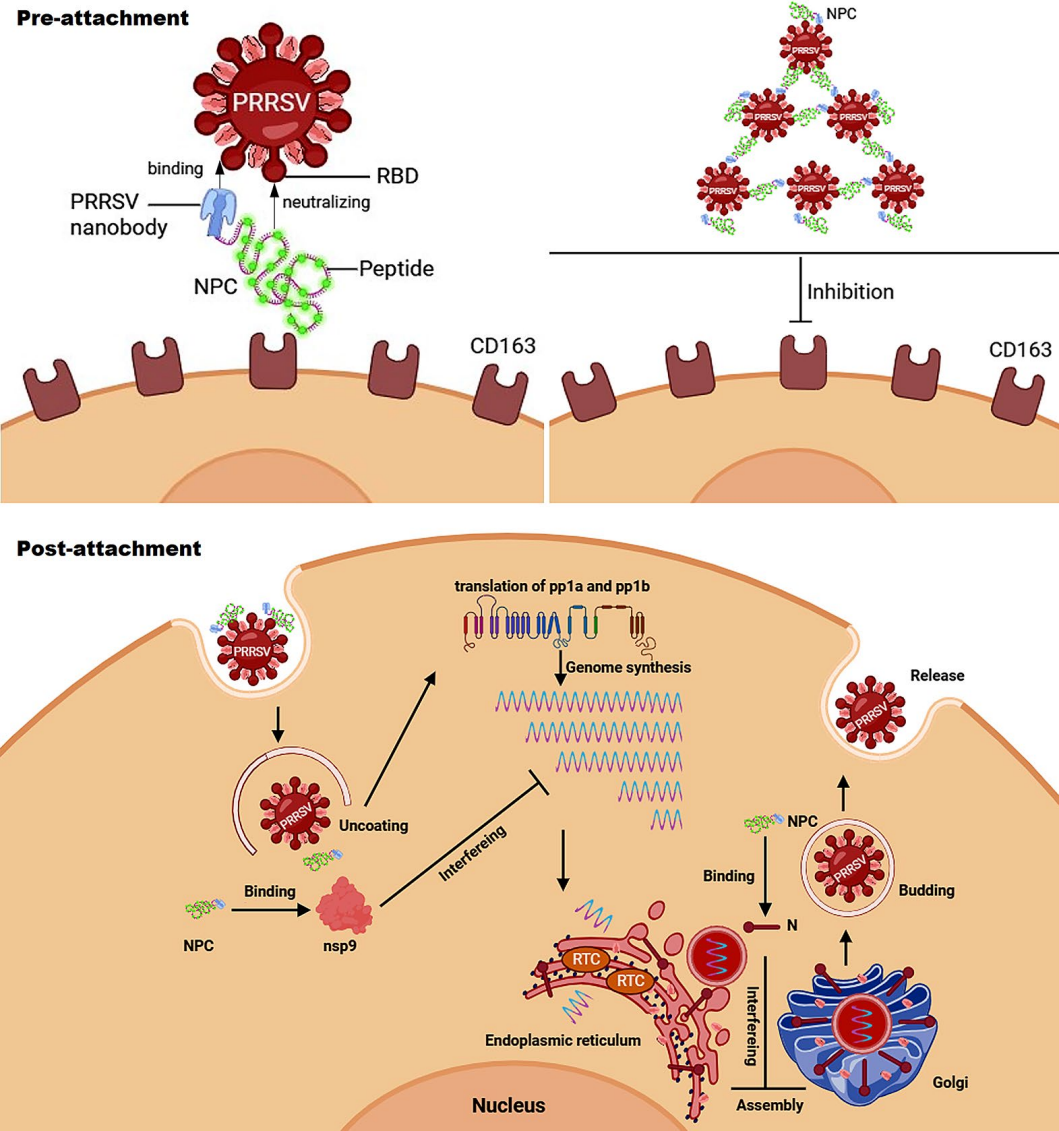
Schematic illustration of NPC neutralization mechanism. NPCs are active for virus inhibition in the stages of pre- and post-attachment. [1] In the stage of pre-attachment, peptides derived from CD163 can block the receptor binding domain (RBD) of PRRSV. Simultaneously, Nbs part in NPCs enhance the affinity of peptides to virions. Due to NPCs can target structure protein, such as N, two components in one NPC molecule may support to catch multiple targets, acting as a linker to connect multiple of virions together for effective neutralization. [2] In the stage of post-attachment, virus enter cells with NPCs. Nbs part in NPCs targeting virus proteins also restrict virus intracellular replication. For example, NPCs with Nbs against PRRSV N/nsp9 exert intracellular antiviral effects by the inhibition on viral assembly and genome synthesis. Figures were created with biorender.com

In this study, we firstly demonstrated that it is available to form a new specific antiviral complex by linking highly targeting nanobody with antiviral peptide. In detail, four NPCs were successfully constructed by using two specific non-neutralizing nanobodies targeting PRRSV N and nsp9, recombining with two antiviral peptides 4H7 or 8H2 derived from CD163 receptor targeting the RBD of PRRSV proteins respectively. Our results showed that all NPCs demonstrated superior viral binding capability and exhibited broad-spectrum inhibitory effects against various strains from different lineages of PRRSV-2, including lineage 1 (HNXX-16 strain (NADC30-like)), lineage 5 (JK-100 strain), lineage 8 (FH-112, JS18-3, HuN4-F112 and JXA1 strains), in a dose-dependent manner, both on PAMs and Marc-145 cells. Mechanistically, as shown in Fig. 8, NPCs can exhibit inhibitory effects via the double insurance. First, NPCs utilize CD163 epitope peptides to interfere with the binding of RBD to CD163 receptor in the pre-attachment stage. Comparing CD163 gene sequences between pig and monkey, the homogeneity is 86.67% (peptide 4H7) and 93.33% (peptide 8H2), individually, leading to a broad antiviral efficiency on PAMs and Marc-145 cells. In addition, the nanobodies in NPC targeting PRRSV structure proteins, such as N, may contribute to catch multiple virion targets with improved affinity relying on the coordination in PRRSV binding with two NPC components. The various functions of NPC will be further explored. On the other side, in the event of that NPCs binding to virus in the post-attachment stage or a sub-neutralization dose of NPC treated, NPCs could entry cells together with PRRSV which survived the pre-attachment neutralization. Upon cell entry, the nanobodies in NPC targeting PRRSV proteins, including N and nsp9, exert their intracellular inhibition individually. Intracellular transfection of NPCs successfully suppressed infection-induced innate immunity response, as evaluated by the level of NF-κB and IFN-β. These results demonstrate that these NPCs have the activity to inhibit intracellular virus replication. N protein has been demonstrated it can block the self-interaction of N protein following viral assembly [22], making NPCs targeting PRRSV N protein effective in both pre- and post-attachment stage. The main function of nsp9 is believed to regulate the activity of viral RNA polymerase [32]. In this study, we have also demonstrated that NPCs targeting nsp9 can inhibit the process of synthesizing long viral RNAs. Besides, PRRSV invasion relies on membrane fusion with cellular lipid structures, and the presence of nsp9 in the lipid structure of viral capsule membrane also moderately influences the internalization process [33], though at a less distribution than N protein. Overall, NPCs exhibit antiviral activity in both the stages of pre-attachment and post-attachment.

By analyzing protein-protein complexes, we preliminary identify the potential regions for NPCs binding. On the one hand, as for specific binding ability, the motif 82TAFNQGA88 of PRRSV N interacts with the motif 8FRPDKY12 of nanobody-N CDR3 domain of NPC-N-4H7/8H2 via forming hydrogens, and the W5 site of nanobody-nsp9 CDR3 domain could be vital for recognizing the motif 627FP628 of PRRSV nsp9. On the other hand, as for neutralizing activity, there exists an interaction forming by hydrogens between the aa K31 and T32 sites of neutralizing domain 4H7 of NPC-N/nsp9-4H7 and the motif 171NLRLTG176 of PRRSV GP2a, and then, a motif 152NAFLP156 of PRRSV GP3 could also form hydrogens to bind with the motif 28SSS30 of neutralizing domain 8H2 of NPC-N/nsp9-8H2. According to the accuracy and confidence results of per-residue confidence scores (pLDDT) and domain position confidence (PAE) of the predicted models, the higher accuracy and confidence were found in the complexes of Fig. 6A and D (S1). However, as for the prediction models of NPCs binding to the RBD of PRRSV proteins, lower PLDDT and PAE indicate uncertainty in the predictions. It should be noticed that our CD163-derived peptides are short linear epitopes. Due to the dynamic nature of loops, low scores could indicate limited data or inherent flexibility of these regions, making them challenging to be predicted accurately. Our previous study has revealed the effectively neutralizing activity of CD163-derived peptides and identified the vital amino acids which play an important role in PRRSV-2 infection of PAMs. The previous results indicated that both mutations in pCD163 PSTI SS1314AA and SRCR6 KT1718NI abolished the ability of monoclonal antibodies to recognize the corresponding region of this receptor [21]. Surprisingly, in this study, consistent with the previous findings, the predicted interaction was indicated between the aa K31 and T32 sites of neutralizing domain 4H7 of NPC-N/nsp9-CDR3-4H7 and PRRSV GP2a. Also, the motif 28SSS30 was found to contribute to bind with PRRSV GP3. It should not be ignored that the consistence between these results and the relevance of our findings despite computational prediction limitations. The analyzed models provide valuable insights into the structural characteristics and potential functional implications of the RBD of PRRSV proteins. In the future, the predicted complex structure models will be confirmed and compared to experimentally determined structures.

Targeting RBD offers several advantages, as the connection between RBD and receptors is the prerequisite for the virus entering host cells and initiating replication [34]. The interruption in the virus binding to host receptors serves as the complete inhibition on infection because viral invasion and replication will never happen. GP2a and GP4 are known to be the key structure proteins mediating PRRSV attachment [35], and the binding of GP5 and M proteins also promote viral entry and invasion through host cell receptors [36]. Besides, GP2a, GP3, and GP4 are considered able to form a multi-protein complex that playing a vital role in viral infectivity and receptor binding [37]. During infection with PRRSV, epitope A of GP5 acts as a decoy, eliciting most of the non-neutralizing antibodies directed to GP5 and delaying the induction of NAs, and making it difficult to identify the precise RBD which could induce NAs [38]. The combination of RBD masking and complexity is the major obstacle of producing NAs of PRRSV. Moreover, PRRSV infection causes ADE due to the insufficient antibody-virus binding at sub-neutralizing dose, leading to the potential virus infection via Fc receptor mediated endocytosis. NPC presented in the study paves the way for the solution of PRRSV ADE. The mimic peptides derived from CD163 receptor directly interact with the RBD of PRRSV proteins and then exert effective neutralization, which is on of the primary neutralizing strategy of NPCs. On the other hand, NPCs can entry into cells together with bound PRRSV in the stage of post attachment, leading to a strong intracellular inhibition on virus replication.

Antiviral peptides, composed of specific sequences and structures of amino acids, are short chain proteins that can specifically interfere with key proteins of the virus. Compared to traditional antiviral drugs, these peptides offer several advantages. Firstly, they can target multiple viral proteins, inhibiting the life cycle of various viruses and displaying broad-spectrum antiviral activity [39, 40]. Secondly, their specific binding properties can reduce toxicity and minimize side effects on host cells [41]. Additionally, antiviral peptides have low risk of inducing drug resistance, as they often target key motifs required for virus life cycle. In the context of HIV treatment, Enfuvirtide (T-20), a synthetic peptide derived from the human immunodeficiency virus (HIV), inhibits viral fusion to host cells, thus disrupting viral replication [42]. Similarly, the HR2P peptide targets Middle East respiratory syndrome coronavirus (MERS-CoV) by inhibiting the fusion between virus and host cells, thus preventing viral entry [43]. Novel peptides have been successfully designed to demonstrate potent antiviral effects by effectively obstructing the interaction between the spike protein of the SARS-CoV-2 and the human ACE2 receptor [44]. These innovative approaches hold promise for the development of improved antiviral therapeutics in the future.

In this study, the effective working concentration of NPCs is about 5µM. A lot of reports of very low concentrations are developed at the nM levels of therapeutic biologics for human diseases [45, 46]. From a veterinary perspective, there can be different in the working concentration of therapeutic biological products between animal and human uses. Many prevalent human viruses, such as SARS-COV-2 and influenza virus, present high infectivity in susceptible cells with a small amount of virus copies. Hence the dose used for animal virus neutralization in cells is usually higher than human virus as more virus copies need to be neutralized. However, NPC is expressed in prokaryotic system while most therapeutic candidates for humans are prepared in eukaryotic systems, which offers the cost-effective advantage for NPC application. Existing studies have shown 20 µM TAT-nanobody exerts antiviral effect against PRRSV in vitro by targeting viral N protein [47]; TAT-Nb6 significantly inhibited the propagation of PRRSV SD16 at 30 µM [48]. These studies have shown that the working concentration of NPCs is within a reasonable range.

Trimmed peptides at small size are testified to be easily delivered in tissues, reducing the incidence of side effects and significantly simplifying the steps of expression and purification. The synthesized NPC here based on the CDR3 structure demonstrates the promising potentiality for its industrial production. Theoretically, replacing the targeting structure with the CDR3 of any nanobody will be effective. Significantly, NPC can be further modified on its existing structure. Alternative neutralizing components, such as peptides derived from RBD of other viruses or monoclonal antibodies specifically targeting the RBD, could potentially replace the CD163 epitope peptides. Furthermore, the results of the animal study confirmed that NPCs inoculated in vivo for 12–24 h sustained PRRSV inhibitory activity in Marc-145 cells. In future, NPC can be potentially modified into nasal sprays to block the spread and infection of PRRSV and serve an innovative platform to minimize the infection of different airborne viruses. The modified NPC can be expressed in large quantities using biological synthesis using *Escherichia coli*, yeast cells, or eukaryotic cells, followed by purification to obtain the final product. After reducing production costs, NPC can be used for the prevention of viral infections on a large scale.

## Conclusion

In summary, our engineered NPCs exhibit excellent antiviral activity against PRRSV, provides a new candidate for PRRSV neutralization and inspires new vaccine targets. This study makes the deepening understanding in interaction machinery between PRRSV and host. It holds significant implications for controlling PRRSV infection in a positive manner. Meanwhile, we hope to contribute new insight to global efforts in other virus diseases prevention and control based on the NPC structure.

### Electronic supplementary material

Below is the link to the electronic supplementary material.

Supplementary Material 1

## Data Availability

The data that support the findings of this study are available from the corresponding author upon reasonable request.
