# Supplementary figures and images for: Nanobody peptide conjugate: a novel CD163 based broad neutralizing strategy against porcine reproductive and respiratory syndrome virus
Source: J Nanobiotechnology. 2024 Jul 2;22:388. doi: 10.1186/s12951-024-02662-7 (PMC11218349; doi:10.1186/s12951-024-02662-7)

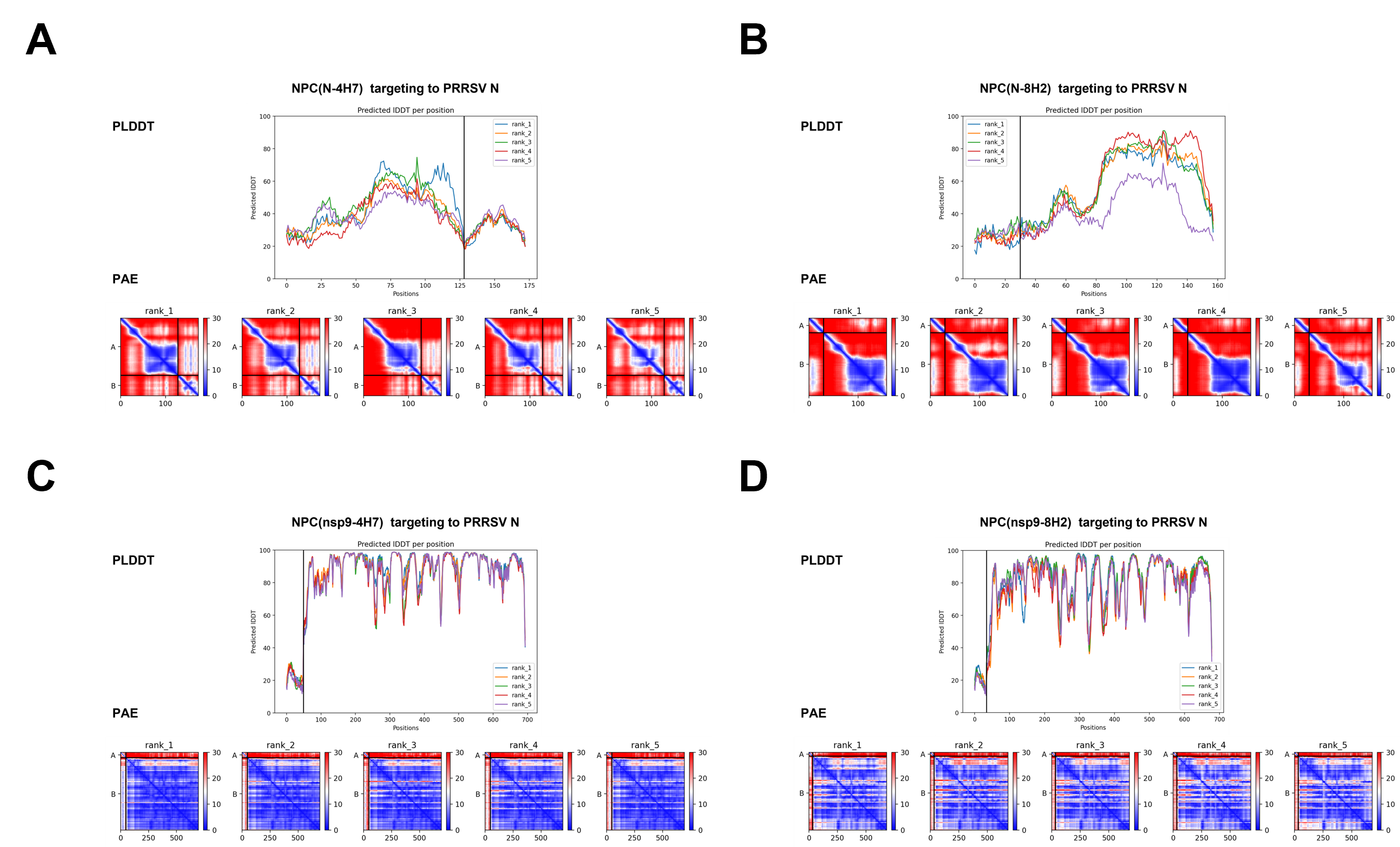

Supplement: Supplementary file 1 — Supplementary Material 1 [file 12951_2024_2662_MOESM1_ESM.tif]
